# Rotavirus acceleration of type 1 diabetes in non-obese diabetic mice depends on type I interferon signalling

**DOI:** 10.1038/srep29697

**Published:** 2016-07-13

**Authors:** Jessica A. Pane, Fiona E. Fleming, Kate L. Graham, Helen E. Thomas, Thomas W. H. Kay, Barbara S. Coulson

**Affiliations:** 1 Department of Microbiology and Immunology, The University of Melbourne at the Peter Doherty Institute for Infection and Immunity, Melbourne, Victoria, Australia; 2 St Vincent’s Institute, Fitzroy, Victoria, Australia; 3 Department of Medicine, The University of Melbourne, St. Vincent’s Hospital, Fitzroy, Victoria, Australia

## Abstract

Rotavirus infection is associated with childhood progression to type 1 diabetes. Infection by monkey rotavirus RRV accelerates diabetes onset in non-obese diabetic (NOD) mice, which relates to regional lymph node infection and a T helper 1-specific immune response. When stimulated *ex vivo* with RRV, plasmacytoid dendritic cells (pDCs) from naïve NOD mice secrete type I interferon, which induces the activation of bystander lymphocytes, including islet-autoreactive T cells. This is our proposed mechanism for diabetes acceleration by rotaviruses. Here we demonstrate bystander lymphocyte activation in RRV-infected NOD mice, which showed pDC activation and strong upregulation of interferon-dependent gene expression, particularly within lymph nodes. The requirement for type I interferon signalling was analysed using NOD mice lacking a functional type I interferon receptor (NOD.IFNAR1^−/−^ mice). Compared with NOD mice, NOD.IFNAR1^−/−^ mice showed 8-fold higher RRV titers in lymph nodes and 3-fold higher titers of total RRV antibody in serum. However, RRV-infected NOD.IFNAR1^−/−^ mice exhibited delayed pDC and lymphocyte activation, no T helper 1 bias in RRV-specific antibodies and unaltered diabetes onset when compared with uninfected controls. Thus, the type I interferon signalling induced by RRV infection is required for bystander lymphocyte activation and accelerated type 1 diabetes onset in genetically susceptible mice.

Type 1 diabetes is a common, chronic disease characterised by immune cell infiltration into pancreatic islets (insulitis), resulting in destruction of the islet β cells that secrete insulin. The diabetic process includes development of immune reactivity to self antigens expressed in islets (autoimmunity), and results in clinical diabetes once insulin levels fall below a critical threshold. Both genetic and environmental factors are associated with type 1 diabetes development^1^. Viruses that are implicated as potential diabetes modulators include enteroviruses, coxsackieviruses and members of the *Rotavirus* genus in the *Reoviridae* family^2^,^3^,^4^,^5^,^6^. Rotavirus infection of children who are genetically predisposed to type 1 diabetes is associated with increased islet autoimmunity and may accelerate progression to diabetes^7^. As in humans, type 1 diabetes development in non-obese diabetic (NOD) mice is affected by genetic and environmental factors and preceded by insulitis development^8^, and these mice are often utilized to investigate virus involvement. Infection of adult NOD mice with Rhesus monkey rotavirus (RRV) accelerates the onset of their type 1 diabetes^9^. Following intestinal infection in these mice, RRV associates with antigen presenting cells, elevates B cell expression of the major histocompatibility complex type I (MHC I), and induces proinflammatory cytokine production by T cells in mesenteric lymph nodes (MLN) and pancreatic lymph nodes (PLN)^10^,^11^. RRV neither infects the pancreas nor induces insulitis^9^. Minimal intestinal immune responses are produced^11^. Regional lymph nodes are thus the likely sites for autoimmune exacerbation^9^,^10^. Pre-existing islet autoimmunity is required for diabetes acceleration by RRV in NOD mice, suggesting that RRV exacerbates rather than causes diabetes in susceptible mice^9^,^12^.

Dendritic cells (DCs) are a group of innate immune cells with functionally distinct subpopulations that fall broadly into two main types, conventional DCs (cDCs) and plasmacytoid DCs (pDCs). The pDCs produce and secrete large amounts of type I interferons following activation, which are crucial to establish the anti-viral state and shape further innate and adaptive immune responses^13^. When stimulated *ex vivo* with RRV, NOD mouse-derived pDCs are sufficient to induce lymphocyte activation, including islet-specific CD8^+^ T cells^14^. This bystander activation depends on signalling through the type I interferon receptor and Toll-like receptor 7, and is heightened in spleen cells from NOD mice over C57BL/6 mice^14^. Activation of pDCs and type I interferon secretion are required for early B cell activation following murine rotavirus infection in non-diabetes prone mice^15^. Thus, pDC activation and type I interferon-mediated bystander activation may contribute to the diabetes acceleration in RRV-infected NOD mice.

Type I interferon signalling is associated with type 1 diabetes onset in NOD mice and humans^16^,^17^,^18^,^19^,^20^. Furthermore, diabetes patients show elevated ratios of pDCs to cDCs^21^. However, as NOD mice lacking a functional type I interferon receptor still progress to diabetes, type I IFN may be redundant for disease development^22^. Since these mice still develop diabetes, they can be utilised in studies of virus-induced diabetes acceleration.

We assessed DC activation following RRV infection and the dependence of diabetes acceleration on type I interferon receptor signalling. RRV-infected NOD mice showed pDC activation and increased type I interferon-dependent gene expression. Without type I interferon receptor signalling, pDC and lymphocyte activation by RRV was delayed and diabetes development was unaltered. Thus, RRV acceleration of diabetes requires type I interferon-dependent responses.

## Results

### RRV rotavirus infection increased the pDC/cDC ratio in NOD mice

**Figure 1:**
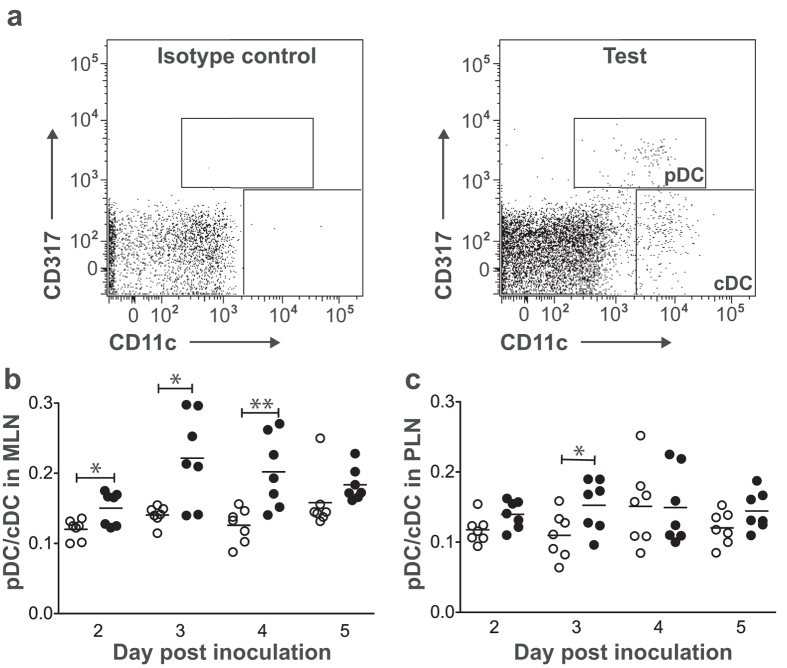
pDC/cDC ratios in lymph nodes of RRV-infected NOD mice. Cells were isolated from adult females given mock inoculum (white circles) or RRV (black circles). (**a**) Flow cytometry plot illustrating DCs within the PLN CD3^−^CD19^−^ cell population. Boxes identify pDCs and cDCs, as indicated on the right panel. The pDC/cDC ratio in MLN (**b**) and PLN (**c**) of each mouse was determined as indicated in (**a**). *p < 0.05; **p < 0.01.

The pDC/cDC ratio is increased in diabetic patients over controls^21^. To determine whether this ratio is elevated in NOD mice in response to RRV infection, pDCs and cDCs were identified by flow cytometry in individual RRV-infected NOD mice and their pDC/cDC ratios determined (Fig. 1a). Compared with mock-infected mice, pDC/cDC ratios were increased in MLN on days 2, 3 and 4 (but not day 5) after RRV infection (Fig. 1b) and in PLN on day 3 (Fig. 1c). As pDC/cDC ratios in PLN were unaltered on days 2, 4 and 5 post infection, RRV infection increased the pDC/cDC ratio in MLN to a greater extent than PLN.

### RRV infection of NOD mice induced pDC activation

**Figure 2:**
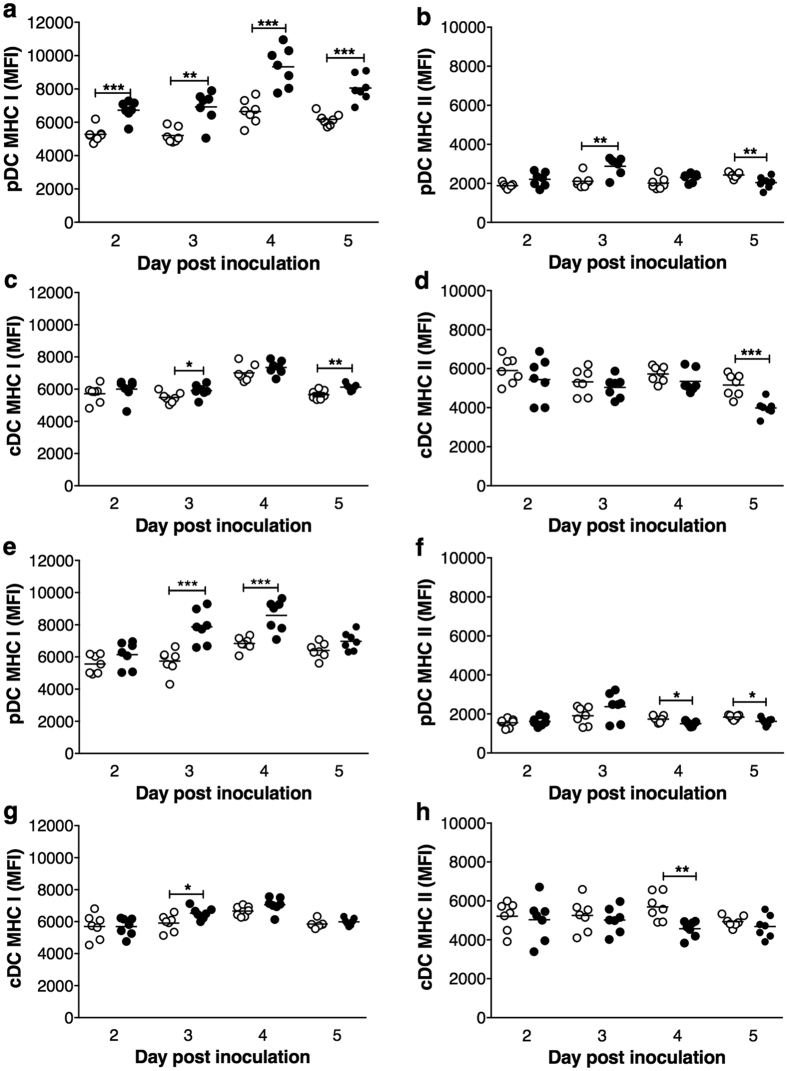
Activated pDC and cDC in MLN and PLN of RRV-infected NOD mice. Cells were isolated from females given mock inoculum (white circles) or RRV (black circles). The mean fluorescence intensity (MFI) of MHC I and MHC II on pDCs (**a**,**b**) and cDCs (**c**,**d**) in MLN and on pDCs (**e**,**f**) and cDCs (**g**,**h**) in PLN was determined by flow cytometry. *p < 0.05; **p < 0.01; ***p < 0.001.

DC activation, as indicated by increased surface expression of classical activation markers MHC I, MHC II, CD80 and CD86, was assessed in regional lymph nodes of mock- and RRV-inoculated NOD mice. In MLN, MHC I levels on pDCs increased at days 2 to 5 after RRV infection (Fig. 2a). MHC II expression on pDCs in MLN increased on day 3 and decreased on day 5 (Fig. 2b). The pDC expression of CD80 and CD86 increased on day 4 (Supplementary Fig. S1). CD86 expression then decreased slightly on day 5. MHC I levels on cDCs in MLN were elevated on days 3 and 5 post infection (Fig. 2c), whereas MHC II expression decreased on day 5 (Fig. 2d). On cDCs in MLN, CD80 expression was unaltered while CD86 expression increased slightly on day 3 then decreased on day 5 (Supplementary Fig. S1).

The pDCs in PLN showed increased MHC I expression on days 3 and 4 post infection (Fig. 2e), decreased MHC II expression on days 4 and 5 (Fig. 2f) and unaltered expression of CD80 and CD86 (Supplementary Fig. S2). CD80 expression also was unaltered on cDCs in the PLN (Supplementary Fig. S2). However, cDCs in PLN showed elevated MHC I on day 3 (Fig. 2g), reduced MHC II on day 4 (Fig. 2h), and a small increase in CD86 expression on day 3 (Supplementary Fig. S2). Splenic pDCs were not activated by RRV infection on day 3, although CD86 expression slightly increased on splenic cDCs (Supplementary Fig. S3). Overall, RRV infection induced pDC and cDC activation in MLN and PLN of adult NOD mice, with pDCs showing greater activation than cDCs.

### Activation of pDCs in infected mice was rotavirus- and mouse strain-specific

**Figure 3:**
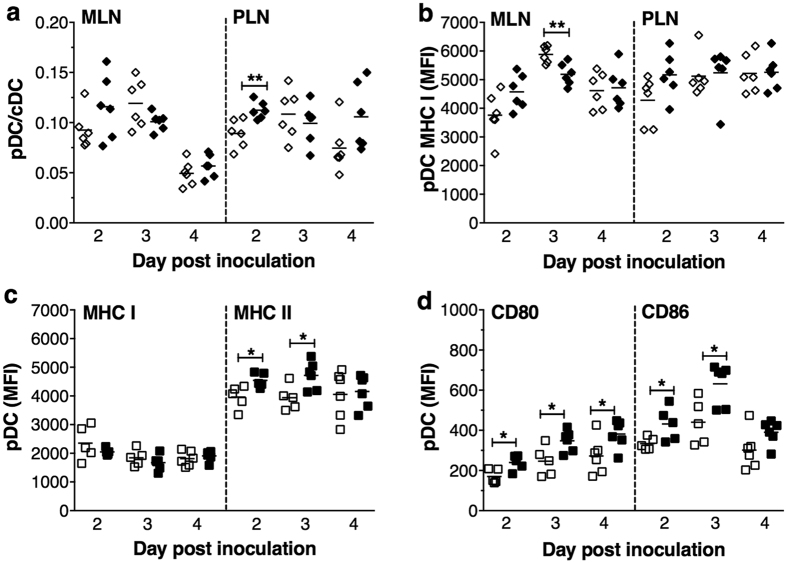
DC activation by rotavirus infection varies by strain of virus and mouse. (**a**) pDC/cDC ratios and (**b**) Mean fluorescence intensity (MFI) of MHC I on pDCs in MLN and PLN of NOD mice given mock inoculum (white diamonds) or CRW-8 (black diamonds). (**c**) MHC I and MHC II and (**d**) CD80 and CD86 levels on pDCs in MLN of C57BL/6 mice given mock inoculum (white squares) or RRV (black squares). *p < 0.05; **p < 0.01.

RRV spreads to NOD mouse MLN and PLN on days 1 to 4 following infection, peaking at day 3^10^. In contrast, infectious porcine rotavirus CRW-8 does not spread to NOD mouse MLN or PLN, and CRW-8 infection does not accelerate diabetes onset^10^. The ability of RRV to accelerate diabetes in mice has been shown to relate to the induction of high serum antibody titers and the RRV gene encoding VP7^23^. CRW-8 shares this VP7 with RRV, but shows several amino acid differences to RRV in VP7, which may help explain the inability of CRW-8 to accelerate diabetes onset^23^. To determine if DC activation in NOD mice varies with the infecting rotavirus strain, CRW-8-infected NOD mice were analysed for DC activation on days 2 to 4 post infection. DC changes were limited to a slight increase in the PLN pDC/cDC ratio on day 2 post infection (Fig. 3a), and decreased MHC I expression on MLN pDCs at day 3 (Fig. 3b). CD80, CD86 and MHC II expression on pDCs was unaltered, and cDCs were not activated (Supplementary Fig. S4). Thus, pDC activation in NOD mice is rotavirus strain-dependent, at least for the two rotavirus strains studied. Our ability to analyse the effects of infection with most other rotaviruses, including human strains, on DC activation is greatly restricted by the inability of these viruses to replicate sufficiently in mice^23^,^24^.

The specificity of pDC activation was determined by analysing DC activation in RRV-infected C57BL/6 mice, which are not diabetes-prone. Infectious RRV was detected in 14% (1/7) of MLN at day 3 post infection (56 f.f.u). No virus was detected in PLN on day 3 or in MLN and PLN on day 5. The pDC/cDC ratio was unchanged in MLN compared to mock-infected mice, and slightly decreased in PLN on day 2 (Supplementary Fig. S5). The RRV presence in MLN was sufficient to induce the upregulation of MHC II, CD80 and CD86 on pDC (Fig. 3c,d). However, MHC I expression remained unchanged. The cDC in MLN, and the pDC and cDC in PLN, showed no increase in any activation marker (Supplementary Fig. S5). Thus, C57BL/6 mice were more resistant to RRV-induced DC activation than NOD mice. The reduced DC activation in both RRV-infected C56BL/6 mice and CRW-8-infected NOD mice compared to RRV-infected NOD mice likely relates to the more limited rotavirus spread to the lymph nodes.

### RRV infection induced type I interferon signalling in NOD mice

**Figure 4:**
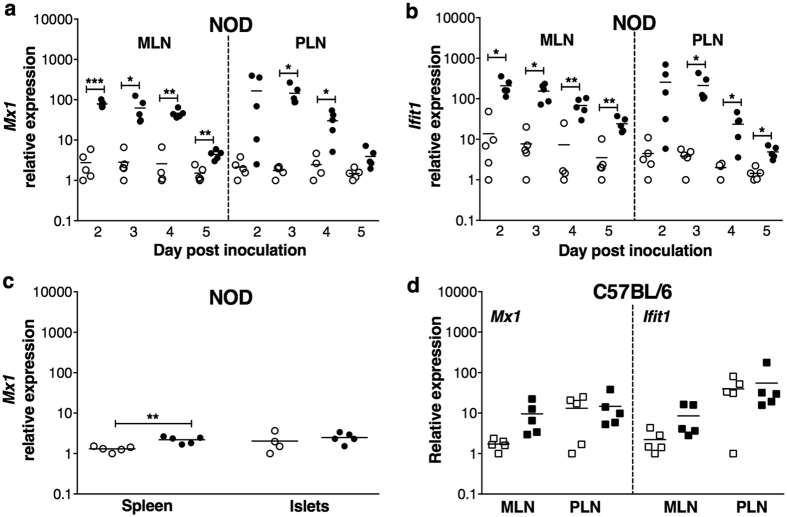
Expression of *Mx1* and *Ifit1* mRNA by NOD and C57BL/6 mice following RRV infection. Cells were isolated from adult female NOD mice given mock inoculum (white circles) or RRV (black circles). *Mx1* (**a**) and *Ifit1* (**b**) mRNA expression levels relative to *β-actin* in MLN and PLN. (**c**) *Mx1* mRNA expression relative to *β-actin* in organs on day 3 post inoculation of NOD mice. (**d**) *Mx1* and *Ifit1* mRNA expression relative to *β-actin* in MLN and PLN cells from C57BL/6 mice at day 3 after mock (white squares) or RRV infection (black squares). *p < 0.05; **p < 0.01; ***p < 0.001.

Since activated pDCs secrete high type I interferon levels, we assessed the expression of type I interferon-stimulated genes in MLN and PLN of RRV-infected NOD mice. *Mx1* and *Ifit1* mRNA expression increased in MLN on days 2 to 5 after infection, compared to controls (Fig. 4a,b). Similarly, *Mx1* and *Ifit1* were upregulated in PLN on days 3 and 4, and *Ifit1* on day 5 also (Fig. 4a,b). *Mx1* levels only slightly increased in the spleen on day 3 (Fig. 4c), reflecting the lack of pDC activation in the spleen (Supplementary Fig. S3). The expression of activation markers on DCs within islets was not directly assessed due to low DC numbers. However, *Mx1* levels in islets were unaltered on day 3, providing evidence that pDCs were not producing type I interferon in islets (Fig. 4c). The absence of RRV from the spleen and islets is a likely reason for the lack of a type I interferon response in these organs. C57BL/6 mice showed a degree of pDC activation due to RRV infection in MLN (Fig. 3c,d) but not PLN (Supplementary Fig. S5). However, neither *Mx1* nor *Ifit1* expression was significantly increased in MLN or PLN of RRV-infected C57BL/6 mice (p > 0.05; Fig. 4d). Overall, RRV infection strongly induced type I interferon signalling in MLN and PLN of NOD but not C57BL/6 mice, and the signalling in NOD mice was mainly localised to these sites.

### RRV presence in lymph nodes of adult NOD.IFNAR1^−/−^ and NOD mice

We analysed the effects of RRV infection in NOD.IFNAR1^−/−^ mice, which produce interferon as for NOD mice but lack a functional receptor for type I interferon. Thus, the signalling cascade that results in expression of interferon-stimulated genes cannot be induced in NOD.IFNAR1^−/−^ mice. Despite the absence of type I interferon signalling these mice still develop type 1 diabetes^22^. As for NOD mice, RRV infection of NOD.IFNAR1^−/−^ mice did not cause diarrhoea. Stool excretion of infectious RRV and/or RRV antigen by NOD.IFNAR1^−/−^ mice was not detected on days 1 to 4 post infection (n = 7/day). Therefore, RRV neither replicated substantially in the intestine nor caused gastrointestinal disease in adult mice in the absence of signalling through the type I interferon receptor.

**Figure 5:**
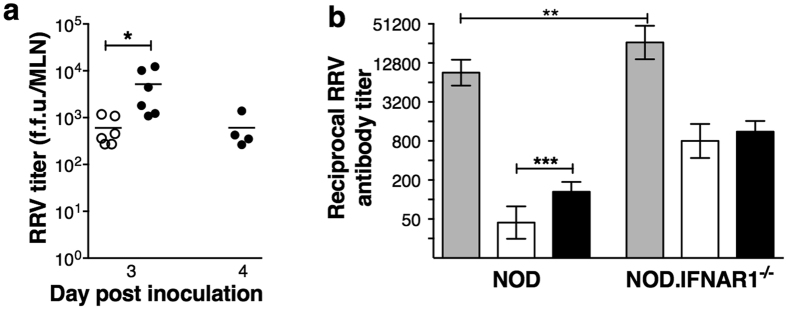
Lymph node RRV titers and serum anti-RRV immunoglobulin levels in NOD and NOD.IFNAR1^−/−^ mice. (**a**) Infectious RRV titers in MLN of NOD (white circles) and NOD.IFNAR1^−/−^ (black circles) mice in fluorescent focus units (f.f.u.) on days 3 and 4 after infection. No virus was detected in mock-inoculated mice (not shown), or in NOD mice on day 4. (**b**) Serum reciprocal anti-RRV titers (geometric mean ± 95% CI) of total immunoglobulin (Ig; grey bars), IgG1 (white bars) and IgG2a (black bars) in NOD (n = 28) and NOD.IFNAR1^−/−^ (n = 21) mice on day 21 post infection. *p < 0.05; **p < 0.01; ***p < 0.001.

Infectious RRV was present in 100% (6/6) of MLN from NOD and NOD.IFNAR1^−/−^ mice on day 3 post infection, with virus titers 8-fold higher in NOD.IFNAR1^−/−^ mice (Fig. 5a). Infectious RRV was cleared from MLN in 100% of NOD and 50% (4/8) of NOD.IFNAR1^−/−^ mice by day 4 (Fig. 5a), and in 100% of NOD.IFNAR1^−/−^ mice by day 5. Infectious RRV was detected in PLN of one NOD.IFNAR1^−/−^ mouse on day 3. Thus, in the absence of type I interferon receptor signalling, RRV persisted for 1 day longer in MLN, and RRV titers in MLN were elevated 8-fold.

### Anti-RRV antibodies in NOD.IFNAR1^−/−^ mice showed no IgG2a bias

Serum anti-rotavirus antibody titers (total Ig, IgG1 and IgG2a) were measured at day 21 after RRV infection as before, when our group has found total antibody levels to reach their maximum^9^,^10^,^23^. NOD.IFNAR1^−/−^ mice developed higher total Ig titers than NOD mice (Fig. 5b). Consequently, NOD.IFNAR1^−/−^ mice showed higher IgG1 and IgG2a levels (p < 0.0001). However, a greater proportion of NOD (75%; 21/28) than NOD.IFNAR1^−/−^ mice (43%; 9/21) produced higher IgG2a than IgG1 titers (Fisher’s test; p = 0.038). IgG2a titers were elevated over IgG1 in NOD but not NOD.IFNAR1^−/−^ mice. Thus, type I interferon signalling is required for production of a Th1-skewed antibody response to RRV in NOD mice.

### Delayed pDC activation after RRV infection in NOD.IFNAR1^−/−^ mice

**Figure 6:**
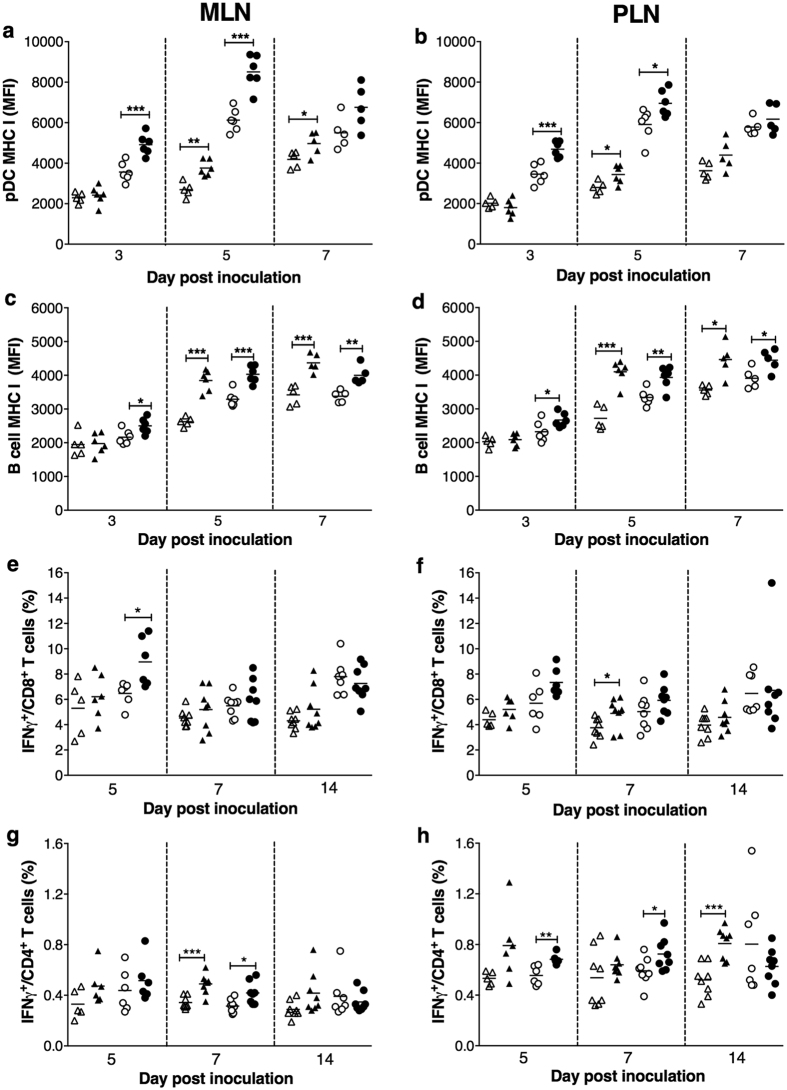
Delayed pDC and lymphocyte activation in NOD.IFNAR1^−/−^ mice. Mean fluorescence intensity (MFI) of MHC I on pDCs in the MLN (**a**) and PLN (**b**) and on B cells in the MLN (**c**) and PLN (**d**) of mock- (white triangles) or RRV-inoculated (black triangles) NOD.IFNAR1^−/−^ mice and mock- (white circles) or RRV-inoculated (black circles) NOD mice is shown. CD8^+^ T cell proportions expressing interferon (IFN)γ in the MLN (**e**) and PLN (**f**) and CD4^+^ T cell proportions expressing IFNγ in the MLN (**g**) and PLN (**h**) in these mouse groups also are given. *p < 0.05; **p < 0.01; ***p < 0.001.

Levels of pDC activation in lymph nodes after RRV infection were determined. MHC I expression was upregulated on pDCs in MLN (Fig. 6a) and PLN (Fig. 6b) on day 3 post infection in NOD, but not NOD.IFNAR1^−/−^ mice. At this time, CD80 and CD86 levels on pDCs in MLN were increased in infected NOD mice, but unaltered in infected NOD.IFNAR1^−/−^ mice (Supplementary Fig. S6). On day 5 post infection, pDC MHC I expression remained elevated in RRV-infected NOD mice in MLN (Fig. 6a) and PLN (Fig. 6b). The pDC expression of CD80 remained slightly increased in MLN but not PLN (Supplementary Fig. S6). Although pDC CD80 levels were unaltered on day 5 post infection in MLN and PLN of RRV-infected NOD.IFNAR1^−/−^ mice (Supplementary Fig. S6), pDC expression of MHC I was elevated in MLN (Fig. 6a) and PLN (Fig. 6b). RRV infection of either mouse strain did not alter pDC expression of CD86 on day 5 or day 7 post infection (Supplementary Fig. S6). On day 7, MHC I levels on pDCs in MLN and PLN of mock- and RRV-inoculated NOD mice were similar. However, pDC expression of MHC I remained elevated in MLN (Fig. 6a) but not PLN (Fig. 6b) of RRV-infected NOD.IFNAR1^−/−^ mice. No mice showed pDC activation in MLN or PLN on day 14 post infection, as evidenced by their unchanged MHC I expression (Supplementary Fig. S7). These data show that lack of IFNAR signalling delayed, but did not prevent, pDC activation in MLN and PLN following RRV infection. It is also of note that pDC MHC I levels in MLN and PLN on days 3 to 14 after mock infection were higher in NOD than NOD.IFNAR1^−/−^ mice (Fig. 6, Supplementary Fig. S7). In addition to RRV-specific effects, it appears that type I interferon receptor signalling also affects pDC activation in NOD mice independently of RRV infection.

### Delayed lymphocyte activation following RRV infection in NOD.IFNAR1^−/−^ mice

RRV infection does not alter the overall proportions of lymphocytes, including B cells and CD4^+^ and CD8^+^ T cells, in MLN and PLN of female NOD mice^9^,^11^. Furthermore, regulatory T cells were only slightly elevated in PLN (not MLN) of NOD mice, at day 7 post infection only (Supplementary Fig. 8). However, RRV infection induces substantial lymphocyte activation in these NOD mice^11^. To determine if this activation requires type I interferon receptor signalling, RRV-infected NOD.IFNAR1^−/−^ mice and NOD mice were analysed.

At day 3 post infection, MHC I expression increased on B cells in MLN (Fig. 6c) and PLN (Fig. 6d) of NOD mice, and CD86 expression increased in MLN (Supplementary Fig. S9). In contrast, B cell expression of MHC I (Fig. 6d) and CD86 (Supplementary Fig. S9) was unaltered at day 3 post infection in RRV-infected NOD.IFNAR1^−/−^ mice. MHC I levels increased at days 5 and 7 post infection on B cells in MLN of NOD and NOD.IFNAR1^−/−^ mice (Fig. 6c). Similar MHC I increases on B cells in the PLN at days 5 and 7 were found in NOD and NOD.IFNAR1^−/−^ mice (Fig. 6d). B cell expression of CD86 and CD80 increased in MLN of NOD.IFNAR1^−/−^ mice but not NOD mice on day 7 (Supplementary Fig. S9). B cells were not activated on day 14 in either mouse strain following RRV infection, as shown by their unchanged MHC I expression (Supplementary Fig. S10). Overall, B cell activation in lymph nodes following RRV infection was delayed but not eliminated in the absence of type I interferon signalling.

T lymphocyte production of the pro-inflammatory mediators interferon-γ (Type II interferon) and tumour necrosis factor following RRV infection of NOD.IFNAR1^−/−^ mice and NOD mice was determined by intracellular cytokine staining. Infection did not affect the interferon-γ levels of T cells in the MLN and PLN of NOD.IFNAR1^−/−^ mice or NOD mice at day 3 (Supplementary Fig. S10). CD8^+^ T cell production of interferon-γ in MLN increased at day 5 after infection in NOD mice only (Fig. 6e). In PLN, RRV-infected NOD mice showed a possible trend for increased interferon-γ production by CD8^+^ T cells on day 5 (Fig. 6f), and interferon-γ levels increased on day 7 in NOD.IFNAR1^−/−^ mice. CD4^+^ T cells in MLN of RRV-infected NOD.IFNAR1^−/−^ and NOD mice showed elevated interferon-γ levels on day 7 (Fig. 6g). In PLN, CD4^+^ T cell production of interferon-γ increased in NOD mice on days 5 and 7 (Fig. 6h). However, CD4^+^ T cell activation was absent from NOD.IFNAR1^−/−^ mice until day 14 post infection. Tumour necrosis factor production from CD4^+^ or CD8^+^ T cells was not increased for either mouse strain. Overall, these data indicate that T cell activation was delayed but not prevented when type I interferon receptor signalling was absent from NOD mice.

### NOD.IFNAR1^−/−^ mice lacked type I interferon-mediated bystander lymphocyte activation

**Figure 7:**
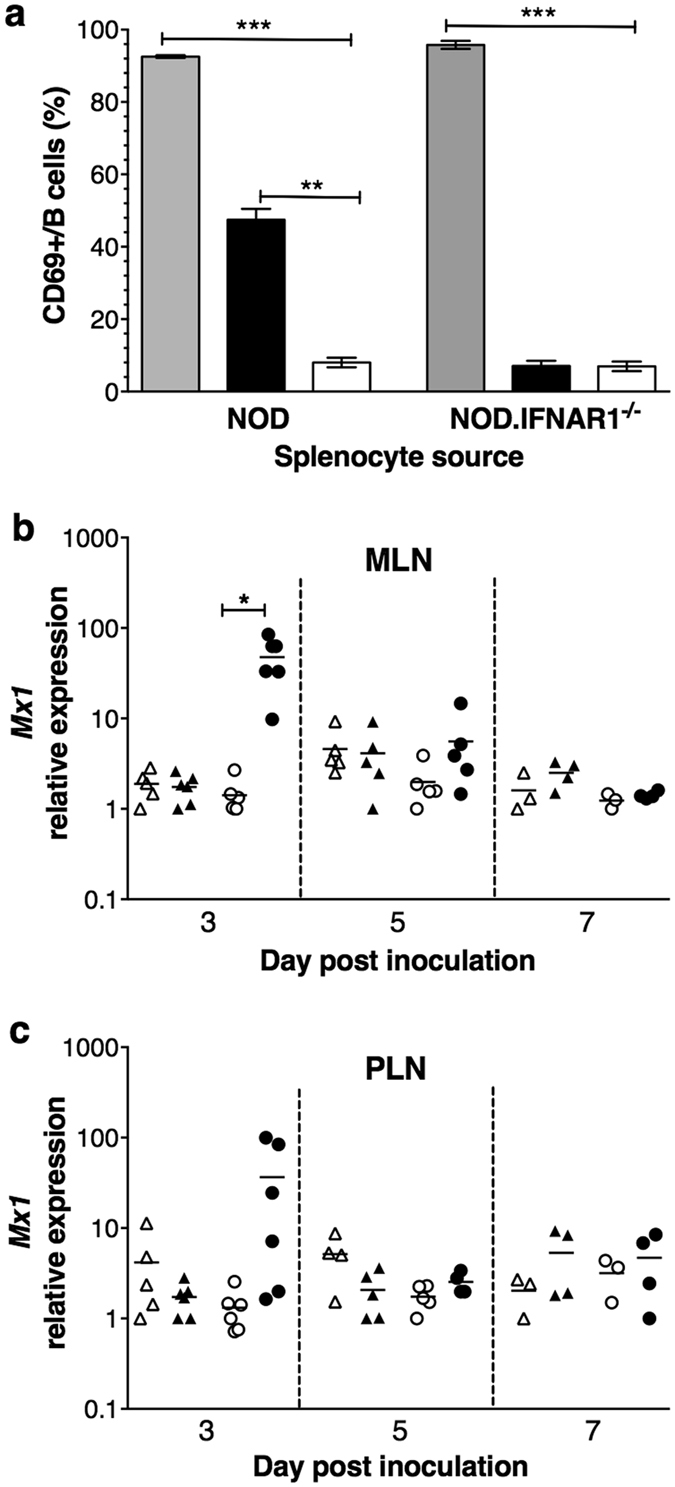
Lymphocyte activation in NOD.IFNAR1^−/−^ mice was not mediated by type I interferon-dependent bystander activation. (**a**) Splenocytes from naïve NOD and NOD.IFNAR1^−/−^ mice stimulated with PMA and Ionomycin C as a positive control (grey bars), cultured with RRV (black bars) or left unstimulated (white bars) for 24 h. Activated B cell proportions were determined. Error bars indicate the SEM of 3 independent experiments. *Mx1* mRNA expression relative to *β-actin* in MLN (**b**) and PLN (**c**) of NOD and NOD.IFNAR^−/−^ mice. Symbols are defined in the Fig. 6 legend. *p < 0.05; **p < 0.01; ***p < 0.001.

*Ex vivo* activation of NOD mouse lymphocytes by RRV requires type I interferon-secreting pDCs^14^. To determine if bystander lymphocyte activation occurs in NOD.IFNAR1^−/−^ mice, bystander B cell activation was measured in RRV-stimulated splenocytes. In control experiments, B cells from NOD mice and NOD.IFNAR1^−/−^ mice were activated by treatment with PMA and Ionomycin C, as expected. In contrast, RRV induced B cell activation in NOD cells alone (Fig. 7a). Therefore, RRV-induced bystander B cell activation depends on signalling through the type I interferon receptor.

To establish if NOD.IFNAR1^−/−^ mice lack type I interferon-dependent gene expression, their *Mx1* mRNA expression was evaluated after RRV infection. As before (Fig. 4), *Mx1* expression increased in MLN of RRV-infected NOD mice on day 3, returning to normal by day 5 (Fig. 7b). The PLN of NOD mice showed similar responses (Fig. 7c). However, lymph node *Mx1* expression was unaltered in RRV-infected NOD.IFNAR1^−/−^ mice (Fig. 7b,c). Thus, NOD.IFNAR1^−/−^ mice lack type I interferon-dependent gene expression, and thus the pDC and lymphocyte activation by RRV in NOD.IFNAR1^−/−^ mice occurs independently of type I interferon-induced bystander activation.

### RRV infection did not accelerate diabetes onset in NOD.IFNAR1^−/−^ mice

**Figure 8:**
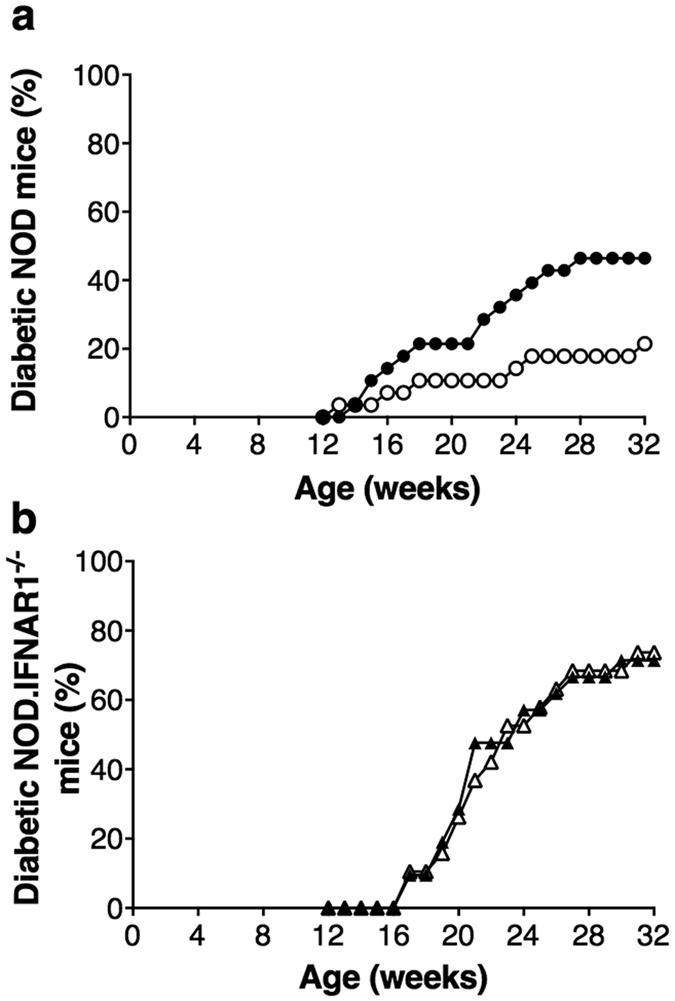
Signalling through the type I interferon receptor is required for diabetes acceleration by RRV in 12 week-old female mice. (**a**) NOD mice given mock inoculum (white circles; n = 28) or RRV (black circles; n = 28) and (**b**) NOD.IFNAR1^−/−^ mice given mock inoculum (white triangles; n = 19) or RRV (black triangles; n = 21) were monitored concurrently for diabetes development.

Mock- and RRV-infected female NOD and NOD.IFNAR1^−/−^ mice were bred, inoculated and monitored concurrently for diabetes onset under identical environmental conditions. After mock inoculation, the overall diabetes incidence was higher in NOD.IFNAR1^−/−^ mice than NOD mice (Fig. 8; p = 0.0003). The diabetes incidence in naïve mice was previously shown to be similar between these strains^22^. The lower incidence of diabetes in our NOD mice may relate to different responses by the two mouse strains to the inoculation procedure and/or the particular environmental conditions of housing. Irrespective of this, RRV infection of NOD mice induced diabetes acceleration in the control experiment (Fig. 8a, p = 0.047) as before^9^. This indicates that the lower than expected diabetes incidence in the control NOD mice is not an issue for the purposes of this study. Notably, diabetes rates were indistinguishable between mock- and RRV-inoculated NOD.IFNAR1^−/−^ mice (Fig. 8b, p > 0.05). Type I interferon signalling thus is required for diabetes acceleration by RRV in NOD mice.

## Discussion

Virus infection may modulate type 1 diabetes development through type I interferon responses. Here we show that RRV rotavirus infection of NOD mice strongly activates pDCs and induces type I interferon-dependent gene expression in MLN and PLN. Importantly, we demonstrate that the diabetes acceleration in RRV-infected NOD mice depends on signalling through the type I interferon receptor. These data provide compelling evidence that rotavirus modulates diabetes by inducing type 1 interferon secretion in regional lymph nodes, and highlight the possibility that diabetes susceptibility is associated with heightened pDC-specific immune responses.

RRV stimulation *ex vivo* of NOD mouse spleen cells induces greater B cell activation than stimulation of spleen cells from C57BL/6 mice^14^. Here, we identify a similar response *in vivo* in RRV-infected mice, with NOD mice showing greater pDC activation and type I interferon-dependent gene expression than C57BL/6 mice. Additionally, pDC activation in C57BL/6 mice remained localised to the MLN, a known site of virus spread^25^,^26^. The dearth of pDC activation in C57BL/6 lymph nodes may be due to the reduced infectious RRV presence in MLN. Alternatively, NOD mouse pDCs may be more responsive to type I interferon-inducing stimuli, as suggested by their heightened secretion of interferon-α when stimulated with CpG^27^. Further analysis is needed to confirm the possible association of heightened pDC activation and function with diabetes susceptibility.

As the NOD mouse genetic background and pre-existing islet autoimmunity are important preconditions for diabetes acceleration following rotavirus infection, it is unlikely that the type I interferon signalling induced by RRV in C57BL/6 mice would facilitate autoimmunity^9^,^10^. Consistent with this, only a very mild, transient hyperglycaemia may be present in association with RRV inoculation of C57BL/6 mice^28^. Rather, the ability of RRV infection to induce type I interferon secretion by pDCs in the lymph nodes appears to be the defining factor associated with diabetes acceleration. This is supported by the inability of CRW-8 rotavirus, which does not accelerate diabetes^10^, to activate pDCs in MLN or PLN.

The activation of pDC following RRV infection was delayed in NOD.IFNAR1^−/−^ mice over NOD mice, suggesting that the early pDC activation in NOD mice is induced by type I interferon. Later pDC responses probably result from direct virus contact in lymph nodes, as pDC activation returns to essentially normal levels by day 7 post infection in both mouse strains, when infectious virus has been cleared. B and T cell activation also was delayed in RRV-infected NOD.IFNAR1^−/−^ mice, implying that early activation of these cells also depends on type I interferon signalling. B cell activation in non-diabetes prone mice at day 3 post rotavirus infection also requires type I interferon receptor signalling, supporting the hypothesis that type I interferon expression is an important part of the immune response to rotavirus^15^. Activated pDCs in NOD.IFNAR1^−/−^ mice secrete type I interferon, but the lack of type I interferon receptor signalling in bystander cells is expected to prevent type I interferon-dependent bystander activation of pDCs and lymphocytes. Indeed, *Mx1* was not upregulated in RRV-infected NOD.IFNAR1^−/−^ mice, indicating that their lymphocyte activation from day 5 post infection occurred independently of type 1 interferon secretion. Although other cytokines possibly might mediate this effect, bystander B cell activation was completely absent following *ex vivo* stimulation of NOD.IFNAR1^−/−^ splenocytes with RRV. Thus, type I interferon expression appears necessary for RRV-induced bystander lymphocyte activation. The small increase in infectious RRV and slightly prolonged presence of RRV antigen in MLN of NOD.IFNAR1^−/−^ mice is consistent with an elevation of rotavirus-specific (rather than islet-specific) immune responses. Supporting this, NOD.IFNAR1^−/−^ mice produced higher overall serum rotavirus antibody titers than NOD mice, indicating a stronger rotavirus-specific B cell response.

It is shown here that type I interferon receptor signalling is required for RRV-induced diabetes acceleration in NOD mice. However, whether rotavirus presence in human MLN and PLN might be associated with type I interferon-dependent responses is unknown. Rotavirus spreads to human MLN^29^ but PLN studies are lacking. Rotavirus stimulation *ex vivo* produces pDC-dependent bystander activation of human B cells^15^. Thus, rotavirus may induce these responses in humans. As the current rotavirus vaccines contain live attenuated viruses^30^, it should be determined if natural infection and/or rotavirus vaccination heightens type I interferon-mediated responses in children at-risk of type 1 diabetes.

The pDC activation and type I interferon responses induced by other viruses also may modulate diabetes development^2^. Reduced MDA-5 expression in NOD mice prevents diabetes acceleration by coxsackievirus B4, and coxsackievirus mRNA in diabetic children is associated with interferon-α^31^,^32^. Additionally, pDC-dependent interferon-α expression is higher in peripheral blood mononuclear cells from diabetic patients than controls in response to coxsackievirus B4 and influenza A, suggesting that high-risk individuals may generate enhanced type I interferon responses upon virus infection^21^. Heightened signalling in pDCs of NOD mice and diabetic patients may help explain why several RNA viruses are linked to diabetes development. Analysis of pDC function after stimulation with a range of RNA viruses would shed further light on this proposal.

## Methods

### Mice

NOD and C57BL/6 mice were obtained from the Animal Resources Centre, Western Australia. NOD.IFNAR1^−/−^ mice were derived as before^22^. Mice were bred and housed in individually ventilated cages under specific pathogen-free conditions, as previously^10^. NOD and NOD.IFNAR1^−/−^ mice were bred and housed concurrently in the same room. All procedures were conducted in accordance with protocols approved by the Animal Ethics Committee of The University of Melbourne. The *Guide for the care and use of laboratory animals* (2011) was followed^33^.

### Mouse inoculation and diabetes monitoring

Rotaviruses were amplified, purified, and infectious titers in fluorescent cell-forming units (f.f.u)/ml and protein concentration determined as before^34^,^35^. 12 week-old female NOD and NOD.IFNAR1^−/−^ mice and 8 week-old female C57BL/6 mice were orally inoculated with virus diluent (mock) or 2 × 10^6^ f.f.u of rotavirus, and monitored weekly for diabetes as relevant, as before^10^,^11^.

### Analysis of cellular activation

Organs were digested with Collagenase A and DNase 1 as before^11^. pDCs (CD3^−^CD19^−^CD11c^low^CD317^+^), cDCs (CD3^−^CD19^−^CD11c^+^CD317^−^) and B cells were identified with antibodies to CD19 (ID3), CD3 (500A2), CD11c (HL3) and CD317 (eBio927). Antibodies to RT1B (OX-6), H2K^d^ (SF1-1.1), CD80 (16-10A1) and CD86 (GL1) also were used. C57BL/6 cells were stained with anti-H2D^b^ (28-14-8) and anti-MHC II (I-A/I-E; 114.15.2). For cytokine measurement, stimulated cells were stained for CD3, TCRβ (H57-597), CD4 (RM4-5), CD8α (53-6.7), IFNγ (XMG1.2) and TNF (MP6-XT22) as before^11^. For regulatory T cell analysis, CD4^+^ T cells were stained for CD25 (PC61) and FoxP3 (FJK-16s) with the Foxp3 Staining Buffer Set (eBioscience).

### Reverse transcription-quantitative real-time PCR (RT-qPCR)

Total RNA was extracted and reverse transcribed from lymph node, spleen and islet cells as before^9^,^10^. PCR was performed using Assay-on-demand kits for *Mx1* and *Ifit1* (Applied Biosystems), with *β-actin* as reference gene.

### *Ex vivo* rotavirus stimulation

Splenocytes were cultured for 24 h in the presence of 100 ng/ml rotavirus, 50 ng/ml PMA and 500 ng/ml Ionomycin C, or left unstimulated, as before^14^. Activated (CD69^+^) B cell (CD3^−^CD19^+^7-AAD^−^) proportions were determined by flow cytometry.

### Rotavirus-specific assays

Homogenized organs and stools were assayed for infectious rotavirus as before^10^. Antibody titers in sera collected at 21 days post inoculation were determined by ELISA using homologous rotavirus and control antigens, as previously^10^.

### Statistical analysis

Student’s *t*-test was used, including Welch’s correction as appropriate, unless otherwise indicated. Log_10_-transformed antibody titers were analysed. Log-rank (Mantel-Cox) analysis was applied to diabetes curves. In Figures, bars represent the mean and SEM unless otherwise indicated, and P values are indicated as: *p < 0.05; **p < 0.01; ***p < 0.001.

## Additional Information

**How to cite this article**: Pane, J. A. *et al*. Rotavirus acceleration of type 1 diabetes in non-obese diabetic mice depends on type I interferon signalling. *Sci. Rep.* **6**, 29697; doi: 10.1038/srep29697 (2016).

## Supplementary Material

Supplementary Information
