# Supplementary material for: Rotavirus acceleration of type 1 diabetes in non-obese diabetic mice depends on type I interferon signalling
Source: Sci Rep. 2016 Jul 13;6:29697. doi: 10.1038/srep29697 (PMC4942798; doi:10.1038/srep29697)
Supplement: Supplementary Information [file srep29697-s1.pdf]

Rotavirus acceleration of type 1 diabetes in non-obese diabetic mice depends on type I interferon signalling

Supplementary Figures S1 to S10

Jessica A. Pane[1], Fiona E. Fleming[1], Kate L. Graham[2,3], Helen E. Thomas[2,3], Thomas W. H. Kay[2,3] and Barbara S. Coulson[1] *

[1]Department of Microbiology and Immunology, The University of Melbourne at the Peter Doherty Institute for Infection and Immunity, Melbourne, Victoria, Australia

[2]St Vincent's Institute, Fitzroy, Victoria, Australia

[3]Department of Medicine, The University of Melbourne, St. Vincent's Hospital, Fitzroy, Victoria, Australia

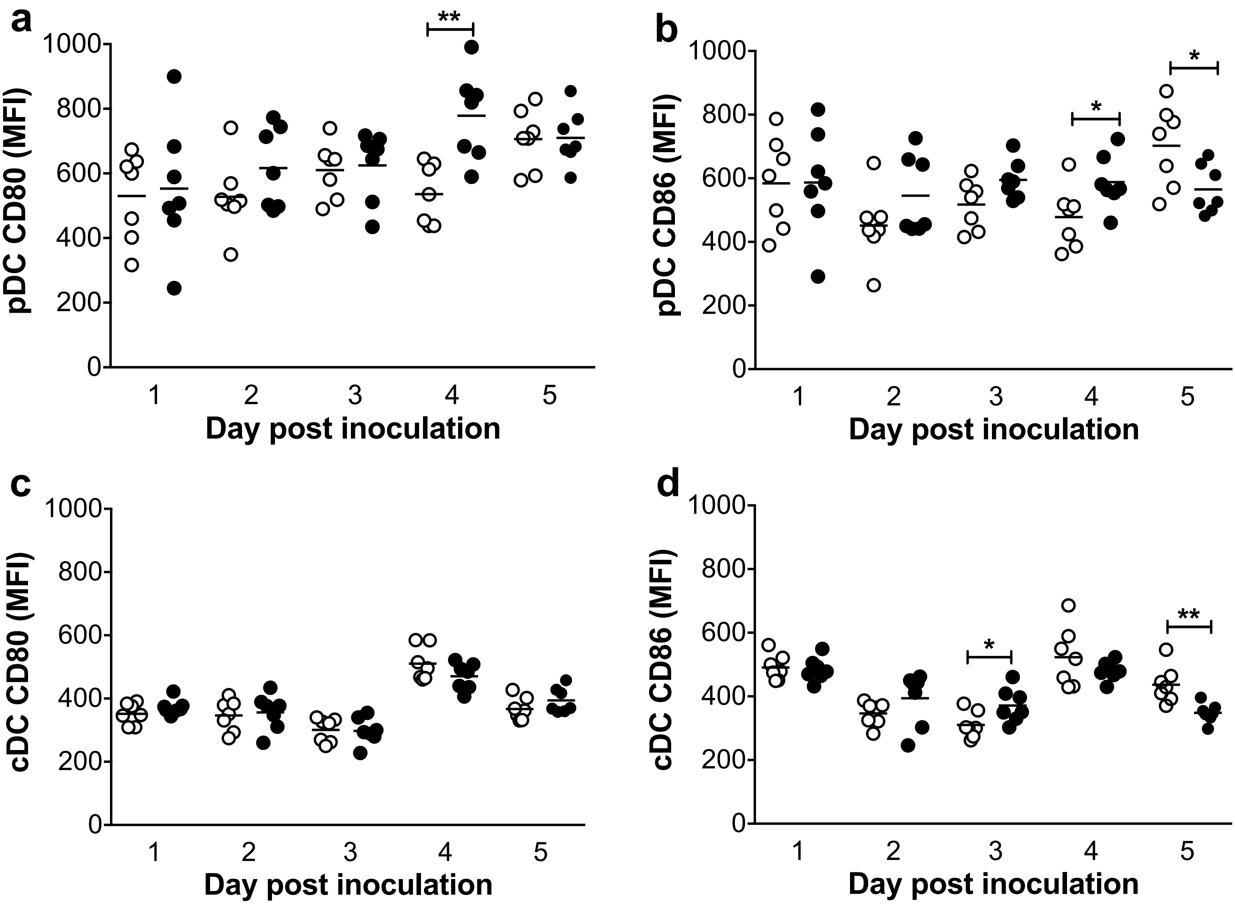

Supplementary Figure S1. Activation of pDC and cDC in MLN of RRV-infected NOD mice. Cells were isolated from females given mock inoculum (white circles) or RRV (black circles). The mean fluorescence intensity (MFI) of CD80 and CD86 on pDCs (**a**, **b**) and cDCs (**c**, **d**) was determined by flow cytometry. *p<0.05; ** p<0.01

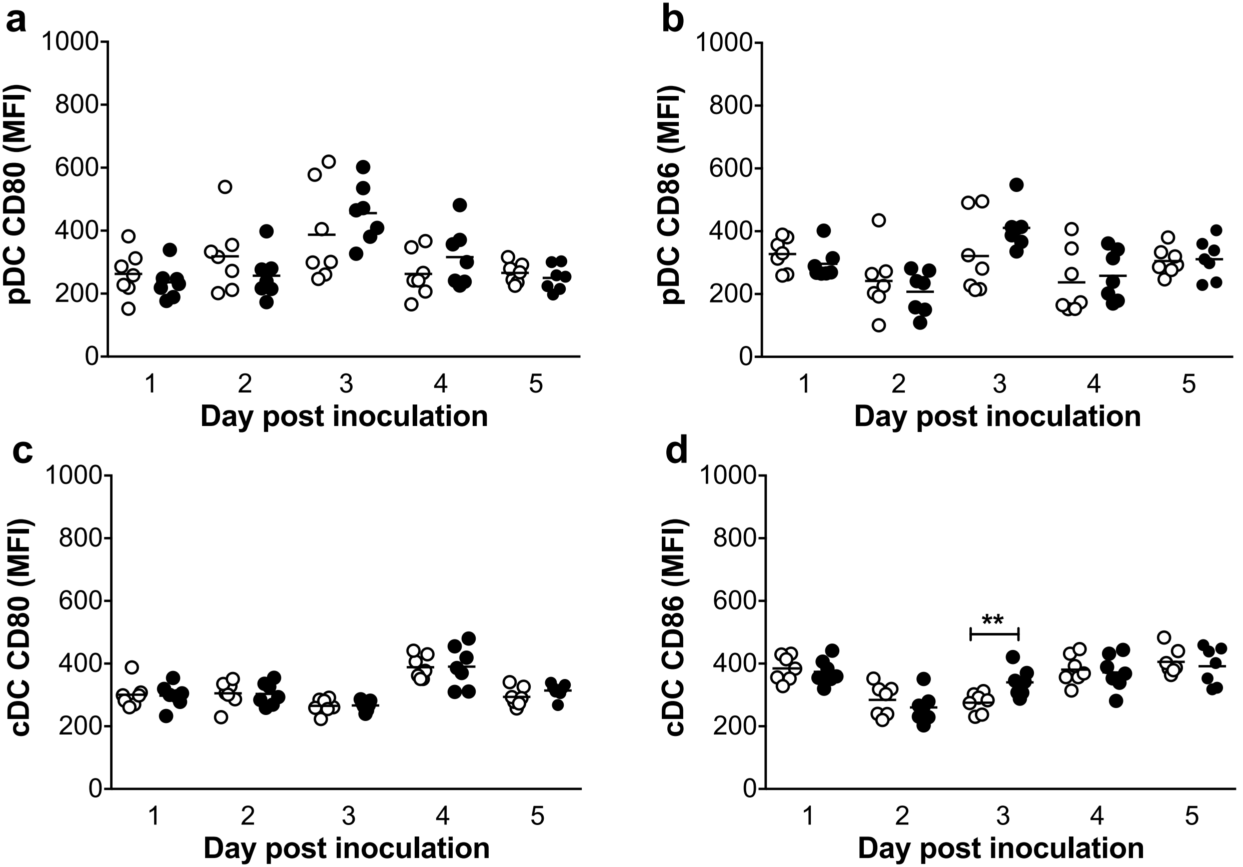

Supplementary Figure S2. Activation of pDC and cDC in PLN of RRV-infected NOD mice. Cells were isolated from females given mock inoculum (white circles) or RRV (black circles). The mean fluorescence intensity (MFI) of CD80 and CD86 on pDCs (**a**, **b**) and cDCs (**c**, **d**) was determined by flow cytometry. ** $p<0.01$

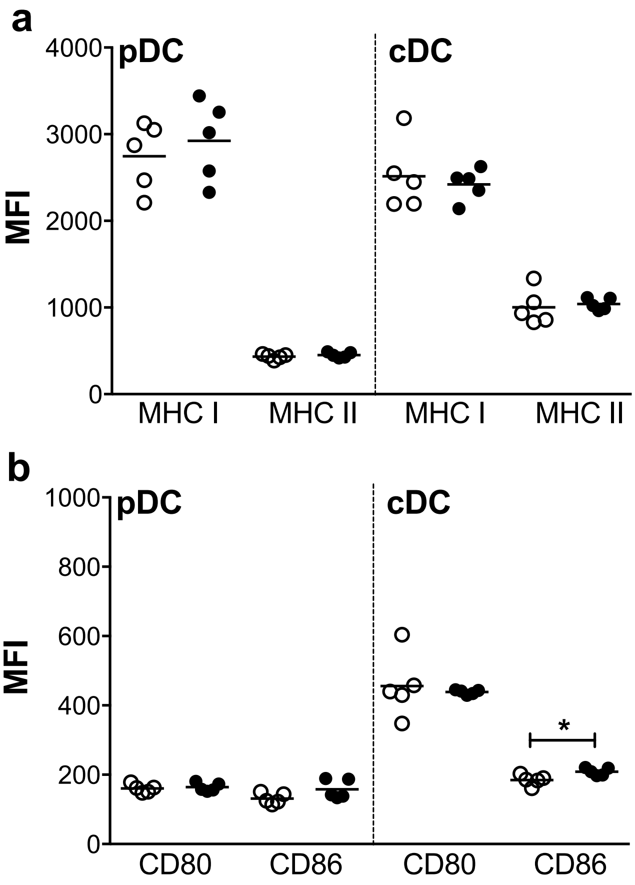

Supplementary Figure S3. Activation of pDC and cDC in the spleen of RRV-infected NOD mice. Cells were isolated from spleens of females given mock inoculum (white circles) or RRV (black circles) on day 3 post inoculation. The mean fluorescence intensity (MFI) of MHCI and MHC II **(a)** and CD80 and CD86 **(b)** on pDCs and cDCs in the spleen was determined by flow cytometry. *$p<0.05$

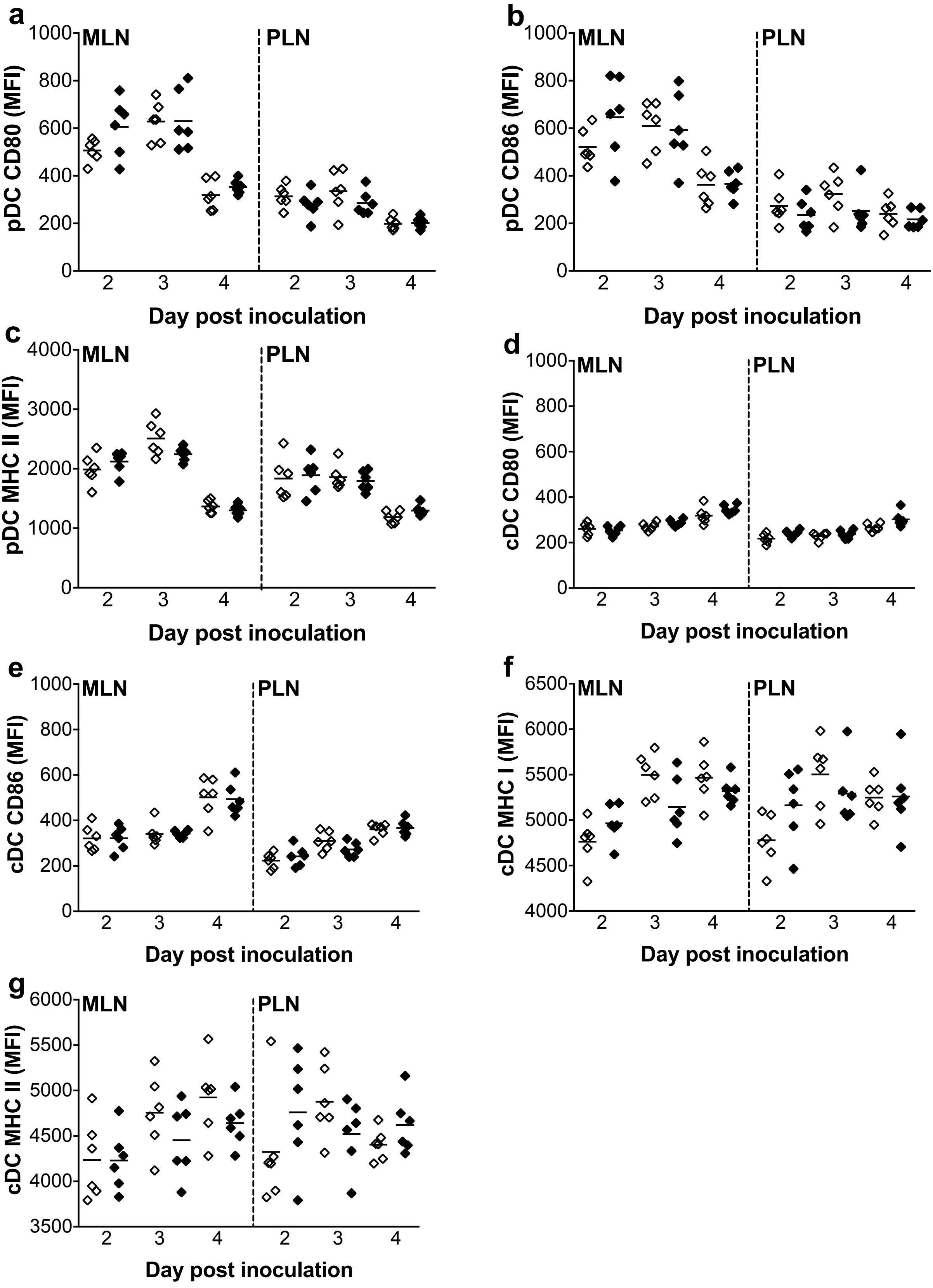

Supplementary Figure S4. Activation of pDC and cDC in MLN and PLN of CRW-8-infected NOD mice. Cells were isolated from females given mock inoculum (white diamonds) or CRW-8 (black diamonds). The mean fluorescence intensity (MFI) of CD80 **(a)**, CD86 **(b)** and MHC II **(c)** on pDCs, and CD80 **(d)**, CD86 **(e)**, MHC I **(f)** and MHC II **(g)** on cDCs in the lymph nodes was determined by flow cytometry.

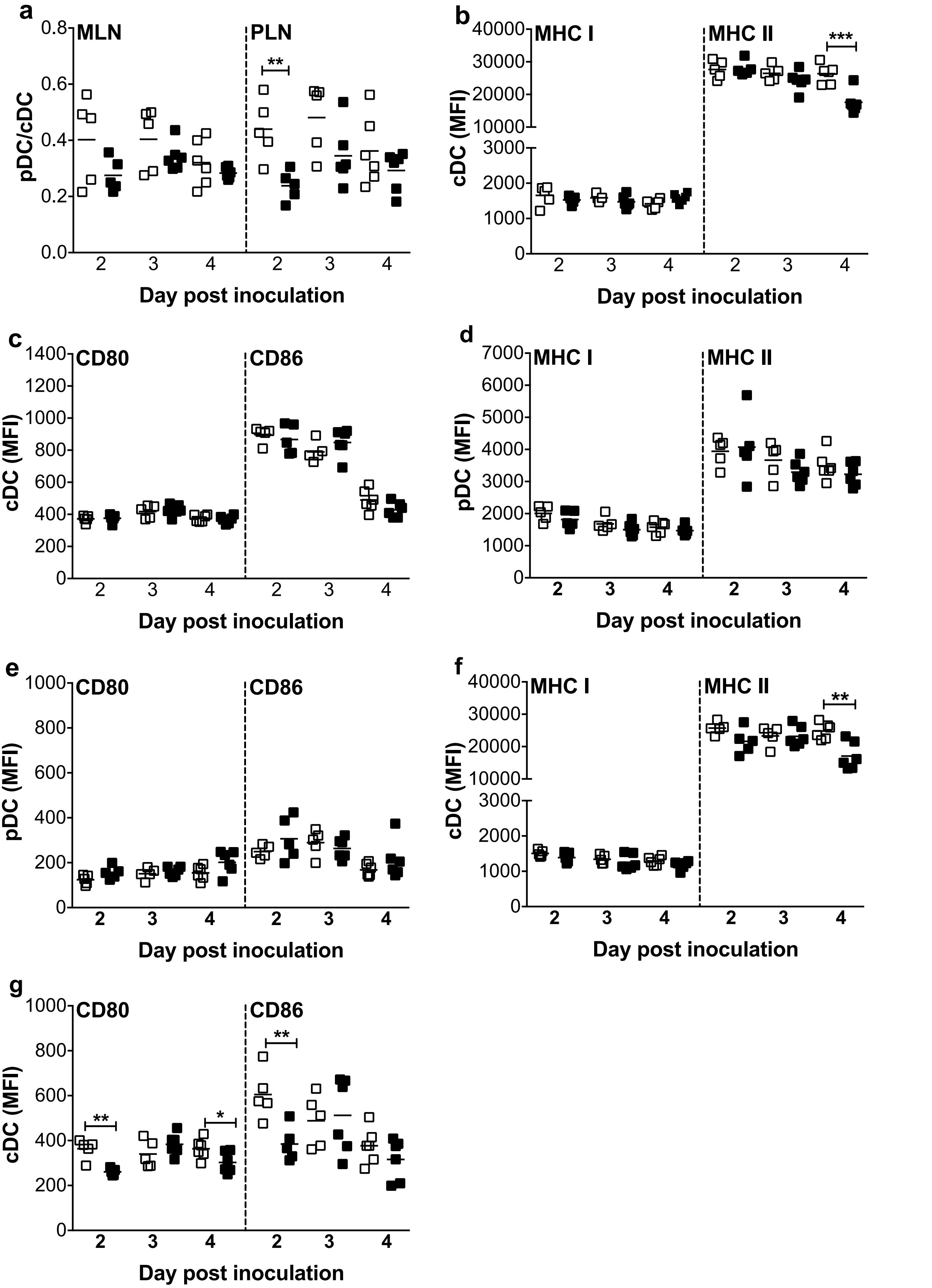

Supplementary Figure S5. Activation of pDC and cDC in MLN and PLN of RRV-infected C57BL/6 mice. Cells were isolated from females given mock inoculum (white squares) or RRV (black squares). **(a)** The pDC/cDC ratio for each mouse. The mean fluorescence intensity (MFI) of MHC I and MHC II **(b)** and CD80 and CD86 **(c)** on cDCs in the MLN was determined by flow cytometry. The expression of MHC I and MHC II **(d)** and CD80 and CD86 **(e)** on pDCs in PLN, and of MHC I and MHC II **(f)** and CD80 and CD86 **(g)** on cDCs in the PLN was similarly determined. *$p<0.05$; ** $p<0.01$; *** $p<0.001$

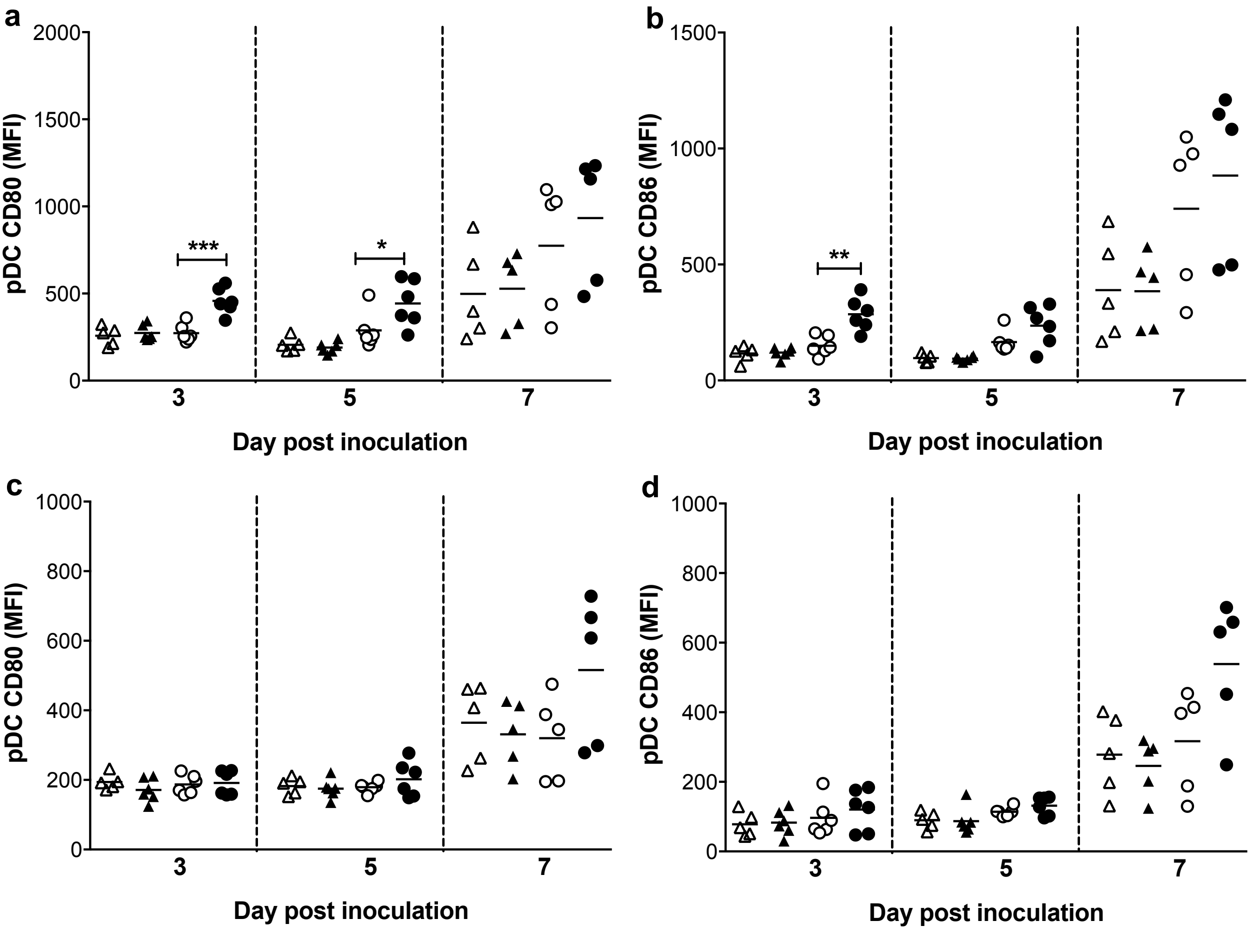

Supplementary Figure S6. CD80 and CD86 expression on pDC in MLN of NOD.IFNAR1$^{-/-}$ mice. The mean fluorescence intensity (MFI) of CD80 and CD86 on pDCs in the MLN (**a, b**) and PLN (**c, d**) of mock- (white triangles) or RRV-inoculated (black triangles) NOD.IFNAR1$^{-/-}$ mice and mock- (white circles) or RRV-inoculated (black circles) NOD mice is shown. *p<0.05; ** p<0.01; *** p<0.001

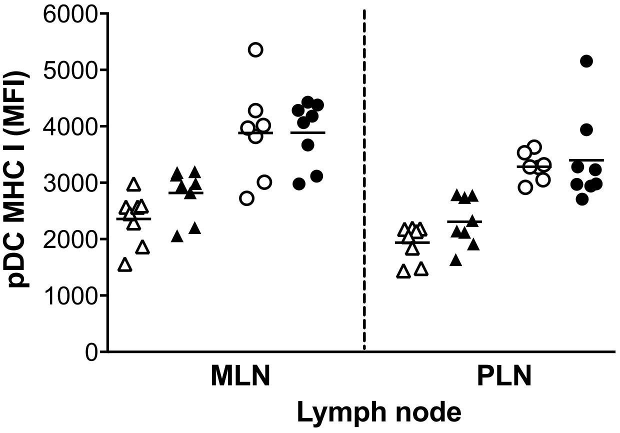

Supplementary Figure S7. Lack of activation of pDC in lymph nodes of RRV-infected NOD.IFNAR1$^{-/-}$ and NOD mice at day 14 post inoculation. Mean fluorescence intensity (MFI) of MHC I on pDCs from the MLN and PLN of mock- (white triangles) or RRV-inoculated (black triangles) NOD.IFNAR1$^{-/-}$ mice and mock- (white circles) or RRV-inoculated (black circles) NOD mice.

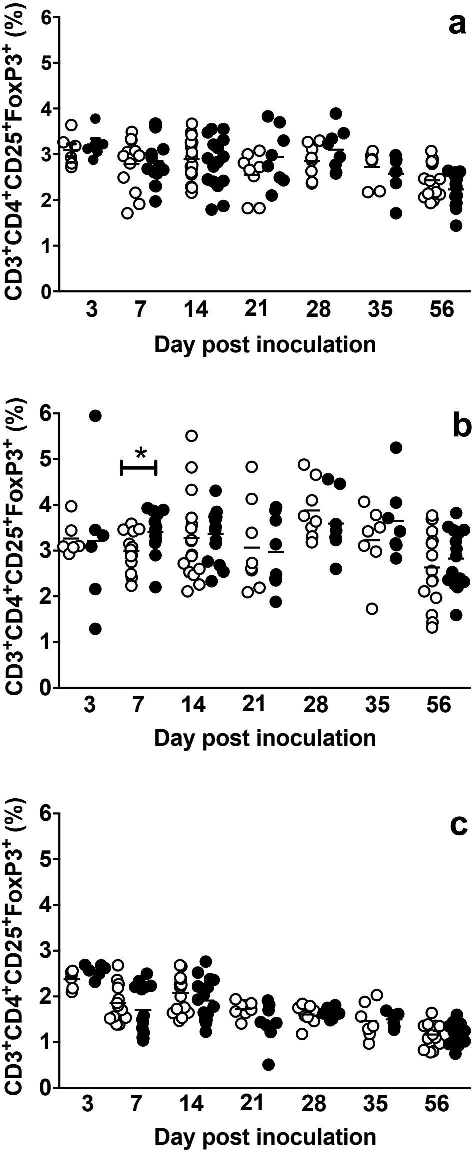

Supplementary Figure S8. **Proportions of regulatory T cells in RRV-infected NOD mice.** The regulatory T cell population ($CD3^{+}CD4^{+}CD25^{+}FoxP3^{+}$) was detected by flow cytometry in cells isolated from (**a**) MLN, (**b**) PLN and (**c**) spleen of groups of adult females given mock inoculum (white circles) or RRV (black circles). * $p < 0.05$

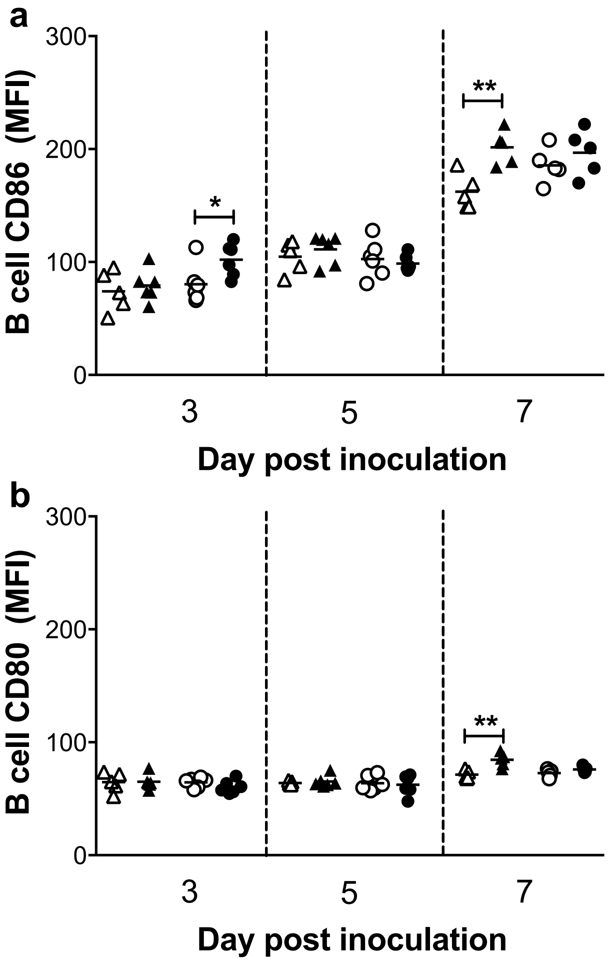

Supplementary Figure S9. Effect of RRV infection on CD80 and CD86 expression by B cells in NOD.IFNAR1$^{-/-}$ mice. Mean fluorescence intensity (MFI) of CD80 (**a**) and CD86 (**b**) on B cells in the MLN of mock- (white triangles) or RRV-inoculated (black triangles) NOD.IFNAR1$^{-/-}$ mice and mock- (white circles) or RRV-inoculated (black circles) NOD mice. *p<0.05; ** p<0.01

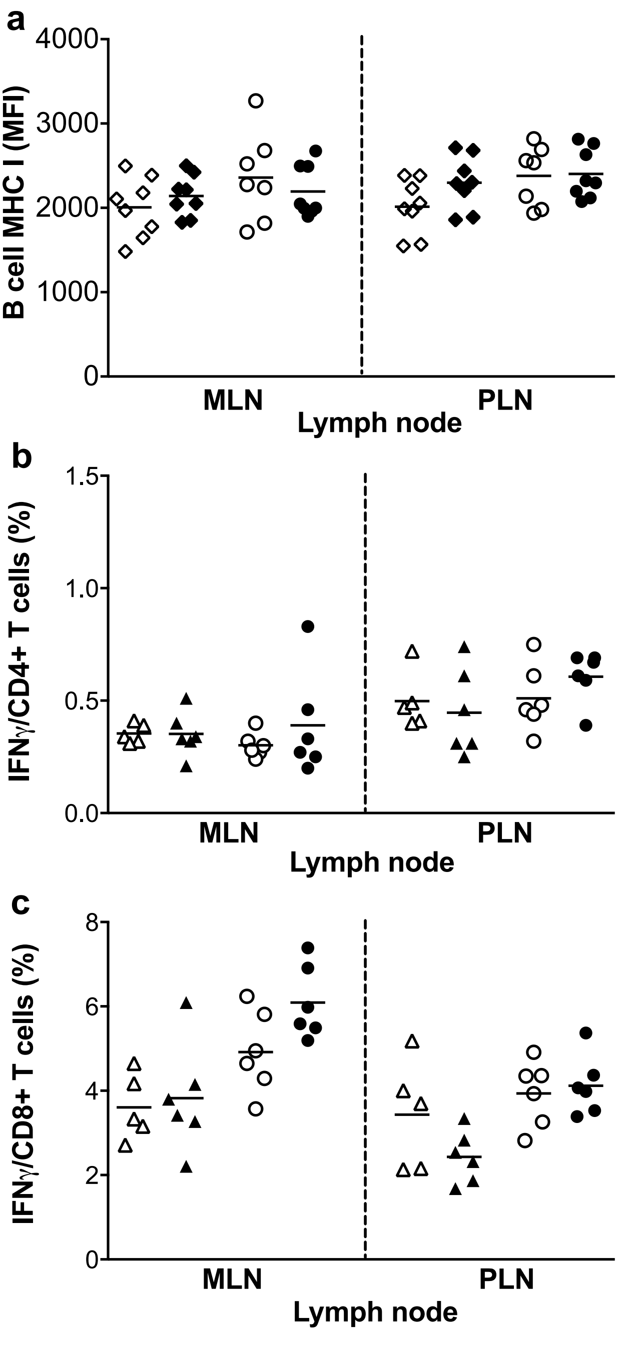

Supplementary Figure S10. Lack of lymphocyte activation in lymph nodes of RRV-infected NOD.IFNAR1$^{-/-}$ and NOD mice at days 3 and 14 post infection. (a) Mean fluorescence intensity (MFI) of MHC I on B cells of mock- (white triangles) or RRV-inoculated (black triangles) NOD.IFNAR1$^{-/-}$ mice and mock- (white circles) or RRV-inoculated (black circles) NOD mice on day 14 post inoculation. The CD4^{+} T cell **(b)** and CD8^{+} T cell **(c)** proportions expressing interferon (IFN)γ in the MLN and PLN at day 3 post inoculation in these mouse groups also are shown.
